# Case Report: Application of accelerated continuous theta burst stimulation in treatment-resistant depression

**DOI:** 10.3389/fpsyt.2025.1615403

**Published:** 2025-10-03

**Authors:** Guilan Sun, Zhongxia Shen, Minmin Wang, Xiaomei Zhang

**Affiliations:** ^1^ Department of Psychiatry, Huzhou Third Municipal Hospital, The Affiliated Hospital of Huzhou University, Huzhou, Zhejiang, China; ^2^ Department of Biomedical Engineering, School of Biomedical Engineering and Instrument Science, Zhejiang University, Hangzhou, Zhejiang, China

**Keywords:** treatment-resistant depression, theta burst stimulation, transcranial magnetic stimulation, accelerated, high-dose

## Abstract

Treatment-resistant depression (TRD) poses a significant challenge in psychiatric practice. While repetitive transcranial magnetic stimulation (rTMS) has emerged as a promising non-invasive neuromodulation technique for TRD, a subset of patients fails to respond adequately to these traditional rTMS protocols. This case report describes the treatment course of a 53-year-old female patient with a complex psychiatric history. Despite initial successful treatment and remission, the patient experienced a relapse of severe depression characterized by sleep disturbances, anxiety, anhedonia, and suicidal ideation. The patient underwent multiple pharmacological treatments, intermittent theta burst stimulation (iTBS) and electroconvulsive therapy (ECT) with limited success over the course of two years. Subsequently, the patient received accelerated continuous theta burst stimulation (a-cTBS) targeting the right dorsolateral prefrontal cortex (DLPFC). Following a-cTBS treatment (18000 pulses each day for 5 consecutive days), the patient showed significant improvement in depressive and anxiety symptoms, as well as in cognitive functions. Remarkable clinical improvement was observed: the Montgomery Depression Rating Scale score decreased from 32 to 9, the Hamilton Anxiety Rating Scale score dropped from 20 to 6, and suicidal ideation decreased from 13 to 5, ultimately disappearing. The outcomes of this intervention suggest that a-cTBS may represent a viable alternative for patients with TRD who do not benefit from existing treatment modalities.

## 1 Introduction

Depression is a common mental health disorder characterized by persistent sadness, loss of interest or pleasure, feelings of guilt or low self-worth, disturbed sleep or appetite, low energy, and poor concentration. While many patients respond well to standard treatments such as antidepressant medications and psychotherapy, some individuals experience treatment-resistant depression (TRD), which is defined as failure to respond to at least two different antidepressant treatments at an adequate dose and duration (1). TRD poses a significant challenge in psychiatric practice, with approximately one-third of patients failing to achieve remission despite multiple treatment trials. Current treatment options for TRD include pharmacotherapy (2–4), and somatic therapies such as electroconvulsive therapy (ECT) (5). While repetitive transcranial magnetic stimulation (rTMS) has emerged as a promising non-invasive neuromodulation technique for TRD, a subset of patients fails to respond adequately to this intervention (6, 7).

Traditional rTMS involves the application of magnetic pulses to specific brain regions for TRD, typically targeting the left dorsolateral prefrontal cortex (DLPFC), to modulate neural activity (8). To optimize stimulation efficiency and patient tolerability, newer protocols such as theta burst stimulation (TBS) have been developed (9). TBS delivers bursts of high-frequency stimulation at a theta rhythm, and can be applied in two main forms: intermittent TBS (iTBS), which generally produces excitatory effects, and continuous TBS (cTBS), which is believed to induce inhibitory effects on cortical neurons. TBS protocols significantly reduce treatment time while retaining therapeutic efficacy.

cTBS is a novel form of rTMS that has shown promise in the treatment of TRD (10). Unlike traditional rTMS, cTBS delivers bursts of stimulation at theta frequency, which is thought to induce longer-lasting inhibitory effects on cortical excitability (11, 12). Recent evidence indicates that continuous theta burst stimulation (cTBS) can rapidly induce neuroplasticity in the adult human brain, with structural changes occurring within hours after a single session. Studies using voxel-based morphometry have demonstrated that cTBS applied to specific cortical regions, such as the anterior temporal lobe, leads to decreased gray matter density accompanied by altered functional activity and connectivity in the targeted networks. These findings suggest that cTBS induces fast-adapting neuronal plasticity mechanisms, including synaptic morphology remodeling and modulation of cortical excitability, thereby producing significant regional synaptic activity changes. Such rapid and activation-dependent plasticity underlies the therapeutic potential of cTBS in modulating dysfunctional brain circuits implicated in treatment-resistant depression (13, 14). However, the efficacy of high-dose accelerated continuous theta burst stimulation has not been fully studied.

## 2 Case presentation

### 2.1 Chief complaints

The patient is a 53-year-old female diagnosed with depression in 2012. Currently, she has been experiencing recurrent symptoms for over two years.

### 2.2 History of present illness

Starting from July 2022, she experienced recurrent depressive episodes and was re-diagnosed with severe depression. Her main symptoms included sleep disturbances, difficulty falling asleep, occasional insomnia throughout the night, excessive worrying, sensation of scalp jumping upon waking, palpitations, chest tightness, low mood, a tendency to cry, inability to experience joy, fatigue, loss of interest in activities, delayed reactions, feeling like a burden to the family, and a history of attempted suicide. The specific clinical intervention data are shown in **Supplementary Materials**.

### 2.3 History of past illness

The patient is a 53-year-old female diagnosed with depression ten years ago. Initially, she was treated with escitalopram 10mg and alprazolam 0.4mg (based on local tablet formulation), leading to a successful recovery and discharge. She remained stable without medication for nine years.

### 2.4 Personal and family history

Denies any family history of mental illness within three generations.

### 2.5 Physical examination

On physical examination, the vital signs were as follows: Body temperature, 36.8°C; blood pressure, 127/72 mmHg; heart rate, 82 beats per min; respiratory rate, 19 breaths per min. There were no significant abnormalities detected upon cardiac and pulmonary auscultation, and the neurological examination yielded negative results.

### 2.6 Mental examination

The patient is alert and oriented to time, place, and person, with intact self-awareness. They present with neat appearance, furrowed brows, and a melancholic facial expression. Their engagement in interaction is still proactive, and their responses are relevant. They exhibit low mood accompanied by anxiety, with coordinated emotional responses. They express pessimistic and negative thoughts, self-blame, and guilt. Additionally, they show diminished willpower, but their insight remains intact.

### 2.7 Laboratory examinations

No abnormality was found in routine blood, levels of serum tumor markers, thyroid function, and urine analyses.

### 2.8 Imaging examinations

The scan sequences encompass transverse T1WIFLair, T2WIFLair, and DWI with a slice thickness of 6mm and an interslice gap of 2mm, along with sagittal T2FSE with a slice thickness of 6mm and an interslice gap of 1mm. The scan outcomes reveal symmetrical cerebral hemispheres, absence of discernible abnormal signals within the brain parenchyma, a clear delineation between gray and white matter, and ventricular, cisternal, and sulcal sizes and shapes that are commensurate with age. The midline structure of the brain is centrally positioned. Head magnetic resonance scan showed no significant abnormalities.

## 3 Diagnosis

An assessment based on the criteria for Major Depressive Disorder (MDD) outlined in the Fifth Edition of the Diagnostic and Statistical Manual of Mental Disorders (DSM-5) was conducted during her inpatient stay. The assessment revealed that the patient met at least 5 out of the 9 criteria for MDD, including persistent feelings of low mood, markedly diminished interest, insomnia, fatigue, feelings of worthlessness, decreased ability to think or concentrate, and recurrent thoughts of death. These symptoms caused severe impairment in her social functioning and could not be attributed to substance use or other medical conditions. The patient has not experienced manic or hypomanic episodes. According to the Maudsley Staging Method (MSM) for treatment-resistant depression, the patient’s multidimensional assessment indicated a severe and chronic course of depression, scoring 10 points on the MSM (15). The Montgomery Depression Rating Scale was used to assess the depressive emotions, while the Hamilton Anxiety Rating Scale (HAMA) was employed for evaluating anxiety-related emotions. The Beck Scale for Suicidal Ideation (BSSI) was used to assess her suicidal thoughts or the risk of suicide.

Combined with the patient’s medical history and therapeutic protocol, the final diagnosis was TRD.

## 4 Treatment

Subsequently, her medication regimen was adjusted to include “Venlafaxine slow-release capsules 150mg, Quetiapine 50mg, Trazodone 75mg, Zolpidem 10mg, and Clonazepam 2mg.” In conjunction with conventional iTBS for 10 days, targeting the left dorsolateral prefrontal cortex, however, her depressive symptoms showed limited improvement.

### 4.1 Accelerated continuous theta burst stimulation

**Figure 1:**
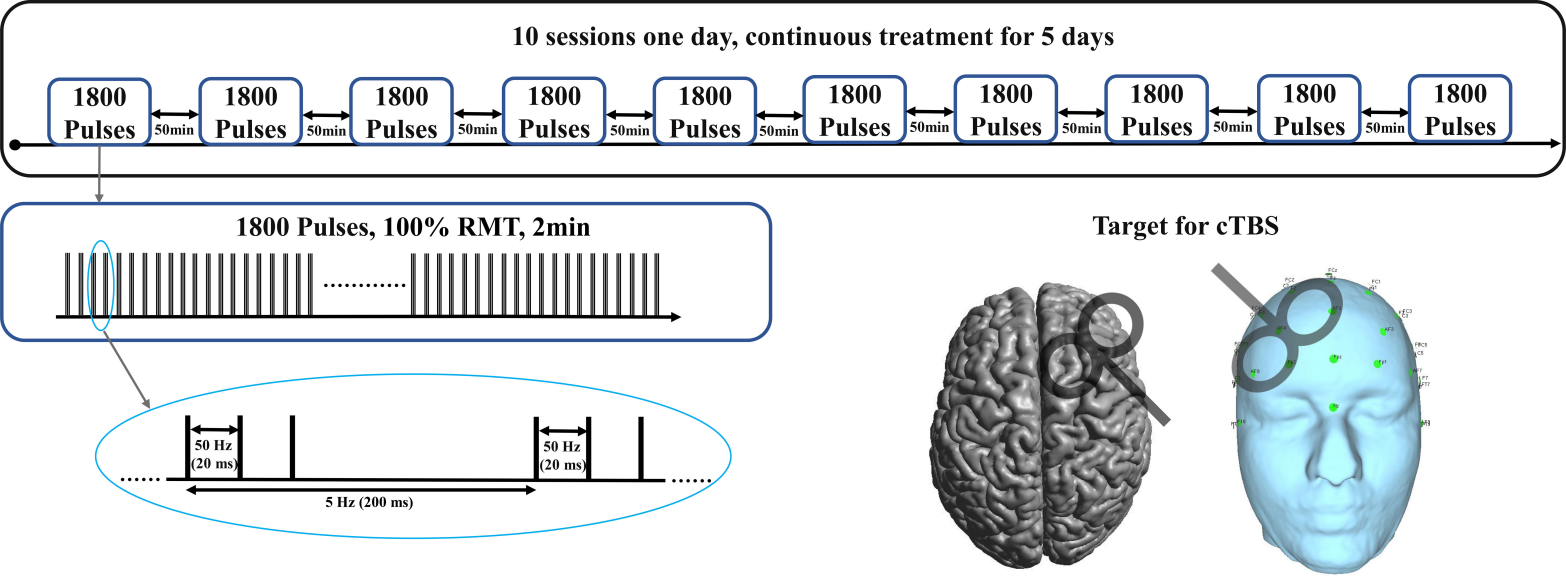
Stimulation protocols of a-cTBS.

Following favorable outcomes with pharmacological and traditional iTBS treatments, we adjusted the treatment strategy to a continuous 5-day course of high-dose a-cTBS as shown in **Figure 1**. The stimulation site was shifted to the DLPFC, with 1800 pulses per session, spaced 50 minutes apart, administered 10 times per day, resulting in a total of 90,000 pulses. Each a-cTBS cycle comprised continuous delivery of 50 Hz triplets at 5 Hz for a total of 1800 pulses. Treatment sessions were administered hourly 10 times a day, totaling 18,000 pulses/day for 5 consecutive days. The stimulation coil was placed at a 45° angle relative to the midline of the brain and located using an EEG positioning cap. Stimulation intensity was set to 100% resting motor threshold (RMT) due to safety considerations (16, 17) and in line with broader clinical guidelines, which was also safe in our previous rTMS studies (18, 19). RMT was determined at the beginning of the treatment and was reassessed daily before each session.

Following consecutive a-cTBS treatments, the depressive mood showed significant improvement. The Montgomery Depression Rating Scale score decreased from 32 to 9, the Hamilton Anxiety Rating Scale score dropped from 20 to 6, and suicidal ideation decreased from 13 to 5, ultimately disappearing.

## 5 Outcome and follow-up

Following consecutive a-cTBS treatments, the patient demonstrated improvement in depressive mood, anxiety, attention, relaxation, and decision-making abilities, but fatigue symptoms persisted. There were no signs of headache during the TMS treatments.

## 6 Discussion

The presented case offers valuable insights into the positive effects of a-cTBS on patients who have shown poor responses to other treatment modalities. The results of this case report suggest that a-cTBS can be a valuable alternative for patients with TRD who have not benefited from traditional treatments such as ECT and iTBS.

The patient in this case had a history of multiple hospitalizations and treatment trials with various medications, including antidepressants, antipsychotics, and anxiolytics, as well as six sessions of ECT. Despite these interventions, the patient continued to experience severe depressive symptoms, including sleep disturbances, anxiety, fatigue, and feelings of worthlessness. After the administration of a-cTBS, the patient showed significant improvement in depressive and anxiety symptoms, as evidenced by reductions in MADRS and HAMA scores. Additionally, suicidal ideation decreased significantly. These findings suggest that a-cTBS may offer a promising treatment option for TRD patients who have not responded to conventional therapies.

The success of a-cTBS in this case could be attributed to its unique mechanism of action (20–22). Unlike traditional rTMS protocol, which delivers high-frequency stimulation, a-cTBS delivers bursts of theta-frequency stimulation, which is thought to have a more potent and longer-lasting effect on cortical excitability (23, 24). This may explain why a-cTBS was effective in this patient where other treatments had failed. Furthermore, the safety profile of a-cTBS appears to be favorable, as the patient did not experience any adverse effects during the treatment period (25). Moreover, the use of a higher dosage of a-cTBS in this case might have contributed to the recovery (26). This suggests that exploring higher doses of a-cTBS could be beneficial for patients who have not responded to lower doses or other treatment modalities. In addition, we targeted the right dorsolateral prefrontal cortex for a-cTBS treatment in this case study, providing an alternative approach to traditional left-sided stimulation in TRD. Several Studies have suggested that right TMS stimulation can be just as effective as left-sided approaches, offering additional options for patients who do not respond well to left TMS stimulation, but further research is needed to determine the optimal dosage and treatment parameters for a-cTBS in TRD, as well as to identify patient characteristics that may predict a positive response to this intervention (27–29).

Since the discovery of the glymphatic system, growing interest has emerged regarding its potential role in psychiatric disorders. Recent evidence suggests that glymphatic dysfunction may be involved in the pathophysiology of various conditions including depression, anxiety, sleep disorders, trauma-related disorders, and substance use disorders. However, clear and direct data remain limited. A recent rapid comprehensive scoping review identified multiple studies highlighting correlations between glymphatic system impairment and psychiatric illnesses such as depression and anxiety, emphasizing the importance of further research using standardized biomarkers and imaging techniques (30). Given the glymphatic system’s critical role in sleep regulation and brain homeostasis, its modulation may represent a novel mechanism through which a-cTBS exerts therapeutic effects, especially considering the common comorbidity of sleep disturbances in treatment-resistant depression. Incorporating this emerging perspective may help to better understand and optimize neuromodulation strategies targeting TRD.

Compared to conventional rTMS, a-cTBS offers advantages in treatment duration and neuroplasticity induction; however, it also presents certain practical and economic considerations. Administering a-cTBS requires daily intensive treatment sessions, often conducted in inpatient or highly coordinated outpatient settings. These logistical demands may pose challenges in terms of clinical infrastructure, personnel availability, and patient adherence. Furthermore, the cost-effectiveness of a-cTBS remains under investigation, especially given the need for specialized equipment and extended daily session times. Future health economic studies are needed to evaluate whether the potentially faster symptom improvement offsets the resource burden of accelerated protocols. In terms of safety and tolerability, no adverse effects were reported in this case. This is consistent with previous reports, which indicate that cTBS and its accelerated forms are generally well tolerated, with side effects comparable to or fewer than standard rTMS protocols. Nonetheless, clinicians should remain vigilant for potential side effects such as transient headache, scalp discomfort, or fatigue, especially in high-intensity stimulation paradigms.

However, there are limitations in this study that should be acknowledged. First, we did not collect pre- and post-intervention imaging data, which could have provided further insight into the brain functional networks affected by a-cTBS. Second, the absence of long-term follow-up data limits our ability to assess the durability of the treatment effects. Although we contacted the patient multiple times after discharge, she consistently reported sustained improvement in mood and functioning. She has since returned to work and continues outpatient follow-up at her local psychiatric hospital, maintaining stability under pharmacological treatment. As a result, no in-person follow-up assessments were performed. Furthermore, given the limited generalizability of single-case reports, Future research should focus on identifying relevant biomarkers to determine which patients are most likely to benefit from specific rTMS treatment modalities. Additionally, more clinical trials are necessary to further explore the efficacy and safety of high-dose a-cTBS, with the ultimate goal of achieving personalized transcranial magnetic stimulation treatments.

## 7 Conclusion

In conclusion, this case highlights the potential of a-cTBS as a novel and effective treatment option for patients with TRD who have not responded to conventional therapies. Further research is warranted to validate these findings and to elucidate the optimal parameters for a-cTBS treatment in this population.

## Data Availability

The original contributions presented in the study are included in the article/**Supplementary Material**. Further inquiries can be directed to the corresponding author/s.
