# Supplementary material for: Case Report: Application of accelerated continuous theta burst stimulation in treatment-resistant depression
Source: Front Psychiatry. 2025 Oct 3;16:1615403. doi: 10.3389/fpsyt.2025.1615403 (PMC12531852; doi:10.3389/fpsyt.2025.1615403)
Supplement: Supplementary file 1 [file Table1.docx]

Table 1 The specific clinical intervention situation

| **Number of hospitalizations** | **1** | **2** | **3** | **4** | **5** | **6** | **7** | **8** |
| --- | --- | --- | --- | --- | --- | --- | --- | --- |
| Hospital stay | 2022.7.12-7.27 | 2022.11.24-12.5 | 2023.1.9-1.29 | 2023.3.13-3.24 | 2023.6.30-7.14 | 2023.8.4-8.19 | 2023.10.20-11.19 | 2024.2.5-3.5 |
| Antidepressant drugs | Milnacipran 75 mg | Milnacipran 100 mg | Escitalopram 20 mg | Escitalopram20 mg, mianserin 30mg | Escitalopram  20 mg | Duloxetine 60 mg | Venlafaxine 225 mg, mirtazapine 15 mg | Venlafaxine  150 mg, trazodone  75 mg |
| Synergist | Tandospirone  5 mg |  | Buspirone 10 mg | Buspirone 10 mg | Buspirone 10 mg | Buspirone 10 mg | Buspirone10 mg |  |
| Atypical antipsychotics | Olanzapine 2.5 mg | Olanzapine 1.25 mg | Olanzapine 7.5 mg | Olanzapine 5 mg | Olanzapine 5 mg | Olanzapine 5 mg | Quetiapine75 mg | Quetiapine 50 mg |
| Physical therapy and psychological therapy |  | rTMS  10 times |  | rTMS  10 times、 transcranial direct current stimulation 10 times | Psychotherapy 2 times、Rtms 14 times | Modified electroconvulsive therapy 6 times、psychotherapy 2 times | Psychotherapy 2 times | Intermittent theta burst stimulation 10 times, accelerated continuous theta burst stimulation 5 days |
| Benzodiazepines and other drugs |  | Alprazolam 0.8 mg, zopiclone  7.5 mg | Clonazepam 2 mg | Clonazepam 2 mg |  | Lorazepam 1.5 mg | Clonazepam 2 mg, zopiclone 7.5 mg | Clonazepam2 mg, zolpidem 10 mg |

RTMS: Repetitive transcranial magnetic stimulation
